# Analysis of Soot Propensity in Combustion Processes Using Optical Sensors and Video Magnification

**DOI:** 10.3390/s18051514

**Published:** 2018-05-11

**Authors:** Hugo O. Garcés, Andrés Fuentes, Pedro Reszka, Gonzalo Carvajal

**Affiliations:** 1 Computer Science Department, Universidad Católica de la Santísima Concepción, Concepción 4090541, Chile; 2 Departamento de Industrias, Universidad Técnica Federico Santa María, Valparaíso 2390123, Chile; andres.fuentes@usm.cl; 3 Faculty of Engineering and Sciences, Universidad Adolfo Ibáñez, Peñalolén, Santiago 7941169, Chile; pedro.reszka@uai.cl; 4 Departamento de Electrónica, Universidad Técnica Federico Santa María, Valparaíso 2390123, Chile

**Keywords:** smoke point, diffusion flame, applied image processing, Eulerian Video Magnification, Phase-based Video Magnification

## Abstract

Industrial combustion processes are an important source of particulate matter, causing significant pollution problems that affect human health, and are a major contributor to global warming. The most common method for analyzing the soot emission propensity in flames is the Smoke Point Height (SPH) analysis, which relates the fuel flow rate to a critical flame height at which soot particles begin to leave the reactive zone through the tip of the flame. The SPH and is marked by morphological changes on the flame tip. SPH analysis is normally done through flame observations with the naked eye, leading to high bias. Other techniques are more accurate, but are not practical to implement in industrial settings, such as the Line Of Sight Attenuation (LOSA), which obtains soot volume fractions within the flame from the attenuation of a laser beam. We propose the use of Video Magnification techniques to detect the flame morphological changes and thus determine the SPH minimizing observation bias. We have applied for the first time Eulerian Video Magnification (EVM) and Phase-based Video Magnification (PVM) on an ethylene laminar diffusion flame. The results were compared with LOSA measurements, and indicate that EVM is the most accurate method for SPH determination.

## 1. Introduction

Combustion processes are in the heart of many modern industrial activities that are key for productive sectors and economic development. For example, in the petroleum, forestry and steel industries, more than 90% of the heat and steam production is based on combustion [1]. At the same time, due to the accelerated pace of industrialization and urbanization in recent decades and omission of their side effects, combustion processes have also become a major contributor to the global greenhouse gas (GHG) emissions [2,3]. An important source of pollution derived from modern combustion processes relates to the release of particulate matter (PM) to the atmosphere, a combustion by-product [4,5]. Recent studies place PM as a major contributor to global warming and climate change, just after carbon dioxide (CO${}_{2}$) emissions [4]. PM alters the radiative properties of the atmosphere and has a negative global effect on temperature and climate. Moreover, PM emissions affect the population’s health, being responsible for respiratory system diseases and the subsequent increase in morbidity and mortality rates [4,6,7]. Consequently, because of increasing public awareness about negative environmental effects and the rise of more strict regulations regarding GHG emissions, there is an imperative need for developing more effective diagnostic and control methods for improving the efficiency of industrial combustion processes.

Recent studies show a direct relation between PM emissions and soot formation in flames [8,9]. Analysis of soot in flames is typically made through *soot propensity analysis*, which denotes the competition between the processes of soot formation ($S_{\mathrm{for}}$) and soot oxidation ($S_{\mathrm{ox}}$) occurring within a flame [10,11]. The most common method for analyzing the sooting propensity of a fuel or a mixture is the Smoke Point Height (SPH) approach [9,12,13], which relates the height of a diffusion flame to an operational point where the process reaches a certain threshold value of the fuel flow rate. Usually, the transition from a non-sooting to a sooting regime surpassing the SPH threshold is the result of an increase in both the $S_{\mathrm{for}}$/$S_{\mathrm{ox}}$ ratio and the residence time of soot particles inside the flame [8]. Once the SPH threshold is surpassed, the flame manifests morphological changes in its tip, which breaks open and shows wings. This marks the moment at which the release of soot particles and emissions of PM from the combustion process increase drastically [14].

The current literature in combustion diagnostics reports two main methods for detecting the SPH: (i) direct visual inspection, and (ii) Line-of-sight attenuation (LOSA) measurements. The quality of the estimations of SPH from direct visual inspection depends on the expertise of the observer for noticing faint morphological variations in the flame that are not always perceptible for the human eye, which leads to high bias and poor repeatibility of the results. In contrast, the LOSA method provides a systematic and robust estimation of the sooting behavior of a diffusion flame; however, LOSA has limited utility in practical combustion systems due to the complexity of the observed phenomena, high sensitivity to signal attenuation, and requirement of especialized and expensive equipment (laser sources, pattern light source, etc.) [10,11,14]. In practice, estimating the SPH of a flame in real-world settings is a challenging task, and current approaches suffer from a high sensitivity to the experimental setup that prevents proper comparison and practical validation of the results.

This paper presents a comparative evaluation of two novel approaches for performing soot propensity analysis using techniques for video magnification over flame images. We propose using Eulerian Video Magnification (EVM) and Phase-based Video Magnification (PVM) to amplify subtle morphological variations in a sequence of flame images, facilitating detection of the point at which wings in the tip of flame appear and release of soot to the atmosphere begins. Using these techniques, we performed sooting propensity analysis on an ethylene diffusion flame using oxygen indexes that ranged from 19% to 35%, and compared the results to reference measurements obtained by LOSA. The experiments validate the effectiveness of video magnification techniques for obtaining quick and in situ estimations of PM emissions using sequences of flame images. These model-free methods have the potential to provide the foundation for low-cost and non-invasive procedures for estimating emissions using cameras that may be already present in typical industrial combustion settings. In addition, with the adequate computational support for fast image processing, the proposed method could overcome the limitations related to the inherent delay in the readings obtained from traditional gas chromatographs and other model-based methods, paving the way for advanced control strategies for combustion process [1,15,16].

The rest of the paper is organized as follows: Section 2 summarizes relevant related work to the monitoring of pollutants emissions in combustion processes. Section 3 summarizes the theoretical background of soot propensity by LOSA and and an overview of video magnification by Eulerian and Phase-based methods. Section 4 describes the proposed experimental setup and methodology. Section 5 reports experimental results and analysis for sooting property using both LOSA and the proposed image-based methods using EVM and PVM. Finally, Section 6 concludes the paper.

## 2. Related Work

The literature reports a fair amount of works dealing with the estimation of pollutants emissions based on data-based models [17,18,19] or first principles models [20]. Unfortunately, any model-based approach depends on the intrinsic error of the model, availability of field sensors to record a referential data set, and the presence of hidden data outliers given by sensor failures or decalibration [21]. As an attempt to compensate for these limitations, some authors propose mechanisms for online model parameters update, incorporating in situ measurements of process variables to estimate the concentrations of pollutants such as NO${}_{x}$ [15,22] and CO [23], and the O${}_{2}$ content of the combustion products [24]. However, despite some improvements in specific applications, the intrinsic limitations of model-based approaches still remain.

Soot propensity analysis is a method for estimating PM emissions from combustion processes by observing the flame, which can overcome the intrinsic delay of conventional direct measurements of emissions using gas chromatographs. LOSA provides a systematic method to perform soot propensity analysis, removing the high-bias of estimations obtained from traditional visual inspection of the flame [10,25]. The LOSA technique obtains soot volume fractions by measuring the attenuation of a beam of known light intensity and frequency as it passes through the flame. The intensity of the light reaching the detector at the opposite side of the flame is attenuated due to the presence of the soot particle cloud within the flame, which scatters and absorbs part of the beam. Using Beer-Lambert’s law, the extinction coefficient is correlated to the soot volume fraction [8,10]. In practice, the LOSA method presents some drawbacks that limit its applicability at industrial scale facilities, including (i) high sensitivity of the method to variations in the refractive index of the flame along the optical path; (ii) strong difficulties in carrying out measurements in non-symmetrical or unsteady flames; (iii) inability to perform measurements when the the soot volume fractions are of the order of ppb or lower; (iv) inability to characterize fuel mixtures typically used in industrial boilers [5,26,27]; (v) requirement of specialized equipment; and (vi) high-computational cost for processing the collected data [10,28]. Note that the method presented in this article can account for variations in the soot volume fractions along the optical path. This can be accomplished for laminar, steady and axisymmetric flames. In the case of turbulent flames, which are typical of industrial settings, the LOSA method can be used under the assumption that the soot volume fraction is uniform along the optical path.

Rapid advances in optical sensors and computational support for data capturing and processing have triggered interest in alternative non-invasive sensing for combustion diagnostics using optical measurements. Recent work reports effective approaches based on optical sensors for identification of conversion zones in a combustion chamber (drying, devolatilization and char oxidation) [29], estimating the radiation emitted by a flame [30,31,32], and reconstructing the temperature of a volumetric representation of a flame [33]. While these methods focus on energy measurements, they do not address directly the challenges associated to reducing the environmental impact of combustion-based processes.

This work describes a setup and methodology for a model-free estimation of the SPH of a flame using digital images. The proposed approach relies on video magnification techniques to amplify subtle morphological changes in a sequence of images. Several works have shown the effectivity of video magnification in applications such as the detection of heart rate and blood pressure by visible or thermal images [34,35], identification of resonant frequencies, damping ratios of a structure and small undesirable movements in a structure [36,37], or calculation of material properties through visual vibrometry [38]. This work builds upon preliminary proof of concept [39], by comparing the error of two different methods for video magnification (EVM and PVM) applied for soot propensity. The comparison of EVM and PVM regards by the analysis of the overall accuracy in the obtained relation between the SPH and the fuel flow rate for soot propensity, through the calculation of average root-mean-square-error, and the confidence interval for a fixed uncertainty, which represents the systematic and random error respectively.

## 3. Proposed Approach for Smoke Point Detection and Background on Video Magnification

In general, combustion processes are controlled by manipulating the flows of fuel and oxygen. If the fuel flow rate is increased while keeping the oxygen flow constant, then the process will reach an operation point at which the soot formation reaction rates within the flame are large enough to overcome the soot oxidation reactions. The smoke point occurs when the threshold fuel flow rate is reached and soot particles flow outside the flame in the form of black smoke. Considering that the heat release and flame height are proportional to the fuel flow rate [13], the SPH method relates the critical point at which the flame starts releasing smoke with morphological changes in the flame

**Figure 1:**
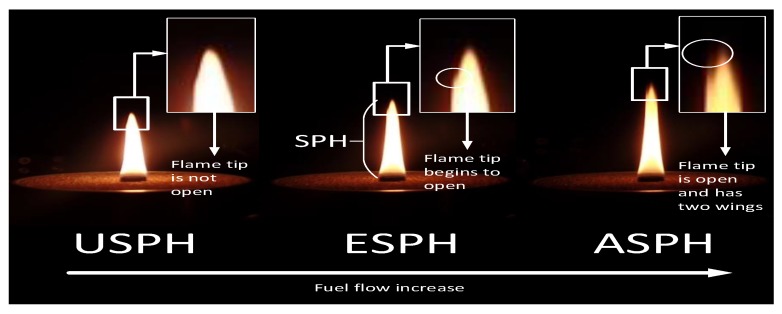
Flame image analysis for soot propensity.

Figure 1 shows morphological properties observed in an axisymmetric diffusion laboratory-scale flame under different levels of fuel flow for a constant oxygen flow. Under SPH (USPH) condition represents the points where the fuel flow rate lies below the threshold value, and the flame has a candle-like shape without releasing smoke. The equal SPH (ESPH) condition corresponds to the critical point when the fuel flow rate reaches the threshold and the tip of the flame shows subtle wings and starts releasing smoke. If the fuel flow rate keeps increasing, then the process enters the above SPH (ASPH) condition, where the wings on the tip become more evident and PM emissions increase drastically [14].

**Figure 2:**
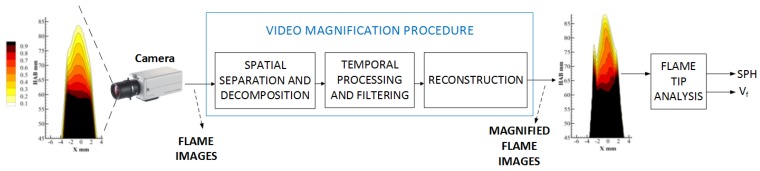
High level representation of the smoke point height detection system based on Video Magnification framework.

Figure 2 shows an overview of the proposed processing flow for performing soot propensity analysis by detecting the smoke point of a flame in a combustion process. The system considers a digital camera that captures a sequence of flame images. The core of the system is the video magnification procedure that takes a stream of images from the camera and produces a new sequence that magnifies variations between consecutive images. We expect the magnified image sequence to reveal information about the subtle morphological changes in the tip of the flame that occur at the smoke point.

To validate the SPH results obtained through video magnification methods, we compare them to estimations obtained from LOSA in the same scenario [8,10], which consists in the calculation of the radially integrated soot volume fraction β within the flame, defined as in Equation (1):

(1) $$\beta =2\pi \int_{0}^{\infty }f_{s}\left(r\right)dr\approx 2\pi \Delta r\sum_{i=1}^{N}f_{s,i}r_{i}$$

where *r* is the distance from the flame’s symmetry axis, i=0,1,…,N−1 is a discretization index for numerical integration, and $f_{s}\left(r\right)$ is the local soot volume fraction calculated as [10]:

(2) $$f_{s}\left(r\right)=\lambda \frac{\kappa_{\lambda }\left(r\right)}{C_{\lambda }}$$

The local soot volume fraction $f_{s}\left(r\right)$ is a function of the absorption function $C_{\lambda }$ and the spectral absorption coefficient $\kappa_{\lambda }$. The absorption function $C_{\lambda }$ is calculated as:

(3) $$C_{\lambda }=\frac{36\pi n_{s}k_{s}}{{\left(n_{s}^{2}-k_{s}^{2}+2\right)}^{2}+4n_{s}^{2}k_{s}^{2}}$$

where $n_{s}$ and $k_{s}$ are the real and complex part of soot’s refractive index, whose values can be evaluated using correlations obtained by Chang and Charalampopoulos correlations [40]. On the other hand, the spectral absorption coefficient $\kappa_{\lambda }$ can be obtained from its relation with the total fraction of light transmitted through the flame (τ), retrieved from the LOSA measurements [41] and the Beer-Lambert’s law according to the following equation:

(4) $$-ln\left(\tau_{\lambda }\right)=2\int_{y}^{R_{f}}\frac{\kappa_{\lambda }\left(r\right)}{\sqrt{r^{2}-y^{2}}}dr$$

where $R_{f}$ is the flame radius.

Finally, the SPH referential value is obtained through the radially-integrated soot volume fraction β, evaluated according to Equation (1) [28]. β is evaluated as a function of the dimensionless axial coordinate (i.e., height above the burner base) $\eta =\frac{zD}{\dot{V}}$, in order to compare the integrated soot volume fraction for different fuel flow rates [28]; where *z* is the axial position where β is calculated, $\dot{V}$ is the volumetric fuel flow rate in sccs, and D is the diffusion coefficient (a value of D=0.156 cm${}^{2}$/s is considered [28]).

### 3.1. Fundamentals of Eulerian Video Magnification

EVM combines spatial and temporal processing to amplify subtle variations in sequences of images using a three-stage approach [42,43]. The first stage corresponds to the spatial decomposition of the original video input into a set of different spatial bands, to recognize the contour of the different shapes in the video [44] and increase the temporal signal-to-noise ratio by pooling multiple pixels [43]. The second stage performs temporal processing on each spatial band separately, by considering a pixel as a time series. Here, a bandpass filter is applied to retain the frequency bands of interest, according to the specific study phenomenon. Then, the filtered bandpass signal is multiplied by a magnification factor α to enhance the desired variations. Finally, in the third stage, the magnified signal is added to the original signal and the spatial pyramid is collapsed to obtain the final amplified output.

I(x,t) is the intensity of a particular pixel *x* recorded in the image at a given time *t*. Since the image is in translational motion, the intensity of a certain pixel can be expressed in terms of an arbitrary spatial displacement function Ω(t):

(5) I(x,t)=f(x−Ω(t))

Then with Equation (5), we could have $I\left(x,0\right)=\tilde{f}\left(x\right)$

We are interested on obtain the time series of pixel variations at each pixel *x* in the form B(x,t)=I(x,t)−I(x,0). Then, by using a first-order Taylor expansion with respect to *x* in Equation (5), the pixel intensity at time *t* can be approximated as:

(6) $$I\left(x,t\right)\approx f\left(x\right)-\Omega \left(t\right)\frac{\partial f(x)}{\partial x}$$

If B(x,t) is the result of applying a broadband temporal band-pass filter on the signal I(x,t) at every position *x* to isolate the term proportional to the displacement function Ω(t), and taking $I\left(x,0\right)=\tilde{f}\left(x\right)$ with $\tilde{f}=f$, then we have:

(7) $$B\left(x,t\right)=-\Omega \left(t\right)\frac{\partial f(x)}{\partial x}$$

This is the first order approximation to the brightness constancy equation in optical flow [42], where the intensity variation at a pixel *x* is the negative of the product between the spatial displacement Ω(t) and the spatial gradient $\frac{\partial f(x)}{\partial x}$. The filtered band-pass signal B(x,t) is subsequently amplified by the amplification factor α and added back into Equation (6), resulting in the processed signal $\tilde{I}\left(x,t\right)$ [42]:

(8) $$\tilde{I}\left(x,t\right)=I\left(x,t\right)-\alpha B\left(x,t\right).$$

where the amplification factor α determining the amplification of subtle color variations, which is obtained according to the specific application. However, an upper bound of amplification factor $\alpha^{*}$ could be obtained [43], which is compatible with accurate motion magnification of the unknown spatial displacement function Ω(t) and cut-off frequency $\lambda_{c}$, given by $\left(1+\alpha^{*}\right)\Omega \left(t\right)=\lambda_{c}/8$ [43]. By combining Equations (6)–(8), we obtain the following result for the pixel intensity:

(9) $$\tilde{I}\left(x,t\right)\approx f\left(x\right)-\left(1+\alpha \right)\Omega \left(t\right)\frac{\partial f(x)}{\partial x}$$

Assuming that the amplified larger perturbation (1+α)Ω(t) can be approximated through a first-order Taylor expansion, then the amplification of the temporally band-passed signal is related to the magnification of tiny motion from the original image source [42]. The processed output in the final stage is then expressed as:

(10) $$\tilde{I}\left(x,t\right)\approx f\left(x-\left(1+\alpha \right)\Omega \left(t\right)\right)$$

where the spatial displacement Ω(t) has been amplified (1+α) times. In summary, the term α defines the amplification factor applied in the video magnification sequence according to the specific application. On the other hand, the term $\alpha^{*}$ describes the upper bound of the amplification factor α and the term (1+α) defines how many times the spatial displacement function Ω(t) is truly amplified.

### 3.2. Fundamentals of Phase Based Video Magnification

PVM modifies local motions in a sequence using a processing flow similar to EVM, but changing the representation from pixel intensities to local spatial phases [42]. In this case, the input image sequence is projected into a basis of complex functions to amplify the phase differences between all independent corresponding basis elements.

Let us reconsider Equation (5), where the intensity of a pixel is expressed in terms of another spatial displacement function, φ(t). We want to obtain a sequence with modified motion as previously defined in Equation (10) for some magnification factor α, but now considering a Fourier basis. Let us rewrite the displaced image profile from Equation (5) as a sum of complex sinusoids as:

(11) $$f\left(x+\varphi \left(t\right)\right)=\sum_{\omega =-\infty }^{\infty }A_{\omega }e^{i\omega \left(x+\varphi (t)\right)}$$

where each band corresponds to a single frequency ω [45].

From Equation (11), the band for a certain frequency $\omega_{0}$ is the complex sinusoidal function $S_{\omega_{0}}$ described as:

(12) $$S_{\omega_{0}}=A_{\omega_{0}}e^{i\omega_{0}\left(x+\varphi (t)\right)}$$

Considering that two consecutive frames in a video sequence are normally slight translations of each other, each coefficient has a slight phase difference in the steerable pyramid representation of motion. Here, the phase term $\omega \left(x+\varphi (t)\right)$ contains motion information. Using the Fourier shift theorem, we can manipulate the motion between frames by modifying the phase of the signal. Assuming that a DC filter removes the DC component of ωx, we can obtain the term $B_{\omega }\left(x,t\right)=\omega \;\varphi \left(t\right)$, which is proportional to the translation. By multiplying the phase shift by α, we obtain an increased phase of sub-band $S_{\omega }$ as:

(13) $${\tilde{S}}_{\omega }\left(x,t\right)=A_{\omega_{0}}e^{i\omega \left(x+(1+\alpha )\varphi (t)\right)}$$

where ${\tilde{S}}_{\omega }\left(x,t\right)$ represents a complex sinusoid motion exactly 1+α times the original value. Finally, by shifting the Fourier coefficients we obtain [42]:

(14) $$f\left(x+\left(1+\alpha \right)\varphi \left(t\right)\right)=\sum_{\omega =-\infty }^{\infty }A_{\omega }e^{i\omega \left(x+(1+\alpha )\varphi (t)\right)}$$

where the two consecutive frames are slight translations of each other, and they have a phase difference given by $\Delta \varphi \left(x,t\right)=\varphi \left(x,t_{1}\right)-\varphi \left(x,t_{0}\right)$, which is amplified by a factor α. Finally, the new frames are reconstructed by multiplying the phase shift and a basis function (usually an amplitude weighted Gaussian kernel [42]). Then, the real part of the new shifted frames is added to get new frames, where the translations and therefore the motions in the flame images are amplified.

### 3.3. Comparing EVM and PVM

It is important to note that the Eulerian perspective of video magnification uses a fixed reference frame and characterizes properties over time at each fixed location [42]. EVM targets the magnification of small variations in consecutive images, and thus certain features that remain mostly stable between images (e.g., height and width of the flame) should remain unaffected. This is a very important property for our specific target application, as SPH analysis requires an accurate value of the flame height at the instant when the flame morphology changes from a non-sooting to a sooting behavior. Unfortunately, since EVM relies on a first-order Taylor expansion, the motion amplification will go out of range if the input motion or the amplification factor is too large [42]. Besides, as a linear motion magnification tool, EVM also tends to amplify the measurement noise. If the original input noise has a high amplitude, then the output image with amplified variations will have a value of $2\alpha^{2}\sigma^{2}$ [42].

PVM amplifies phase differences rather than pixel intensities, which presents two main advantages: (i) it supports larger amplification factors than EVM, (ii) PVM is less sensitive to noise, because the noise amplitude is not amplified [42]. Unfortunately, PVM presents oversmoothing effects that can shape white noise into a false motion signal. Besides, PVM recovers tiny motions at frequencies lower than the temporal Nyquist frequency of the field camera. Thus, if motions are too fast, such as in the case of the flame images, only an aliased version of the motion will be amplified [42].

## 4. Methodology

### 4.1. Acquisition and Analysis of Flame Images

As shown in Figure 2, the processing flow starts with the acquisition of a video sequence from a CCD camera. The recorded videos are processed using EVM and PVM to amplify subtle variations in the flame tip, with the objective of determining if the flame corresponds to either a regime under the smoke point height (USPH) or to a regime equal or above smoke point height (ESPH or ASPH). To amplify the desired elements in the flame video, we need to tune some parameters of EVM and PVM that include the amplification factor α, the cutoff frequency $\lambda_{c}$ according to the range of frequencies where α is applied, and the video frame rate $f_{S}$ provided by the camera. To amplify only subtle variations of the flame tip, we use a narrow frequency band that normally spans from 2 to 4 Hz, in agreement with the laminar conditions of the flame. The frame rate $f_{S}$ is 42 frames per second for the used camera. In general, the cutoff frequency $\lambda_{c}$ depends on the specific application; for soot propensity analysis of laminar flame images, cutoff frequency is set as $\lambda_{c}=20$ Hz, according to the usual flame flickering values. The amplification factor α is set to $\alpha_{0}=20$ and flame images for experimental conditions around the SPH are measured. We repeat this procedure until the morphological changes of interest in the flame images are successfully amplified. If necessary, the amplification factor $\alpha_{0}$ could be increased.

The next step after setting the parameters for video magnification is the SPH analysis. For the image acquisition and SPH analysis, we first run a large sweep with a large variations in fuel flow rate to determine an approximate neighborhood of the fuel flow rate corresponding to the SPH. Next, we perform iterative sweeps with smaller variations around the vicinity of the candidate fuel flow rate to more accurately determine the flow rate at which the flame tip first breaks out and the wings appear. The recorded flame height and fuel flow rate at the onset of the sooting behavior correspond to the soot propensity for the particular experimental conditions.

### 4.2. Experimental Setup

**Figure 3:**
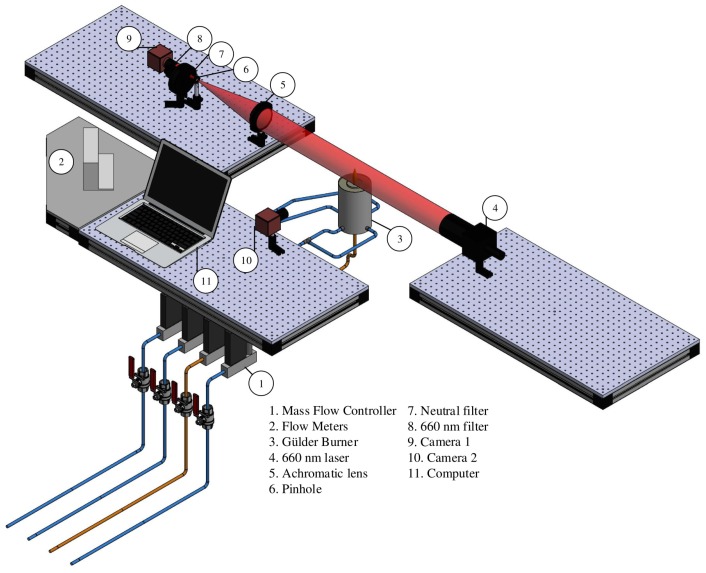
Experimental set-up and target (Gülder) burner detail.

Figure 3 depicts the experimental setup used in this study, which corresponds to an adaptation of the one presented in [10] for measuring soot volume fractions using LOSA. We extended the setup using an additional CCD camera to capture flame images to analyze them using EVM and PVM. By relating a sequence of images to a given LOSA measurement, we can compare and validate the experimental results obtained through video magnification methods.

To generate flames under controlled operational conditions, we use a Gülder burner to obtain a co-flow axisymmetric laminar diffusion flame, which has a negligible variability if the fuel and oxidizer flow rates remain constant. Gülder burners are widely used by the combustion community, as they allow users to perform controlled and repeatable experiments [46,47,48,49].

As a fuel, we use ethylene (C${}_{2}$H${}_{4}$) with a flow rate between 1.3333–5.0000 cm${}^{3}$/s (equivalent to 80–300 sccm). We use ethylene as fuel for the combustion, because of their high soot production for a given oxygen index [11], and high sensibility to the oxidizer composition [50]. Moreover, the pathways and reactions in the soot formation of ethylene flames are well known and featured by the combination of resonantly stabilized radicals that have a well know shared $C_{5}$ side structure [51], which entails the highest soot production.

For a certain oxygen index and SPH testing, we vary the ethylene flow rate $m_{C_{2}H_{4}}$ within the range given by $m_{C_{2}H_{4}\min}$ and $m_{C_{2}H_{4}\max}$. The flow rates of nitrogen $Q_{N_{2}}$, oxygen $Q_{O_{2}}$ and air flows $Q_{\mathrm{air}}$ are set to obtain a specific oxygen index OI%, and to define an experimental condition to carry out the soot propensity analysis. The oxygen index is defined as the oxygen concentration in the full oxidizer flow described in $Q_{\mathrm{total}}$ [10]. For each OI between 19% – 35%, we tested nine fuel flow rates around the smoke point [10]. We control the ethylene and the oxidizer flow rates using Brooks Instrument Inc. digital thermal mass flow controllers with an error of $\pm 1.67\times 10^{-3}$ cm${}^{3}$/s (equivalent to ±0.05%). Table 1 summarizes the different experimental testing conditions for the diffusion flame, which depend on the ethylene and oxidizer flows.

To obtain the reference measurements for SPH using LOSA, we use an Andor Luca R EMCCD camera (shown as Camera 1 in Figure 3), with a pixel resolution of 1004×1002, spectral range between 400–1100 nm, and pixel ratio of 8 μm × 8 μm. A 600 nm diode laser pulsating at 1 Hz and 1500 mA generates a 100 mm diameter beam that passes through the flame and is concentrated by a 300 mm focal achromatic lens. A band pass interference filter with a peak transmission wavelength at 660 nm (10 nm at FWHM) was mounted in front of the camera. During each experiment, an external pulse generator makes the diode laser pulse at 2 Hz. When calculating the referential SPH by LOSA, we obtain 100 frames for each experimental condition to reduce measurement distortion by the electronic noise of the optical instruments. The laser pulsation generates modulated images of absorption and emission from soot particles as proposed in [41]. The second camera (Camera 2) is a 16-bit monochromatic CMOS Photonfocus, with a pixel resolution of (1038×1038) and pixel ratio of 60 μm × 60 μm respectively, to measure the flame images used in the video magnification with EVM and PVM Finally, the setup considers the use of neutral density filters and to reduce noise.

## 5. Experimental Results and Analysis

### 5.1. Referential Soot Propensity Analysis by LOSA

**Figure 4:**
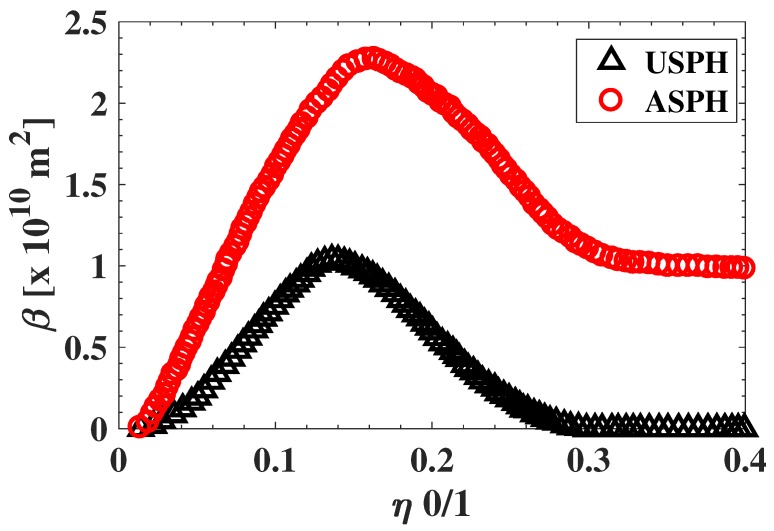
Integrated soot volume fraction for a flame under the smoke point height (USPH), and above smoke point height (ASPH) at OI 21%.

To obtain reference values for SPH analysis using LOSA, we calculated the integrated soot volume fraction (β) using Equation (1). To calculate β, we evaluate soot volume fraction profiles at different flame heights above burner (HAB) for each of the fuel flow conditions in Table 1. Figure 4 shows the calculated β as function of the normalized height fuel flow η for different fuel flow rates and OI = 21%. In particular, the plot shows the results of the integrated soot volume fraction for a flame USPH and ASPH, where a sooting flame is verified if the soot volume fraction β does not reach zero value when η increases and approaches the flame height. Otherwise, if the flame does not release soot, then β approaches zero when η increases. This analysis is repeated for the tested oxygen indices between 19% and 35% as shown on Table 1 to obtain referential SPHs and fuel flow rates at SPH $V_{f}$ for each experimental condition.

### 5.2. Soot Propensity Analysis Using EVM and PVM

**Figure 5:**
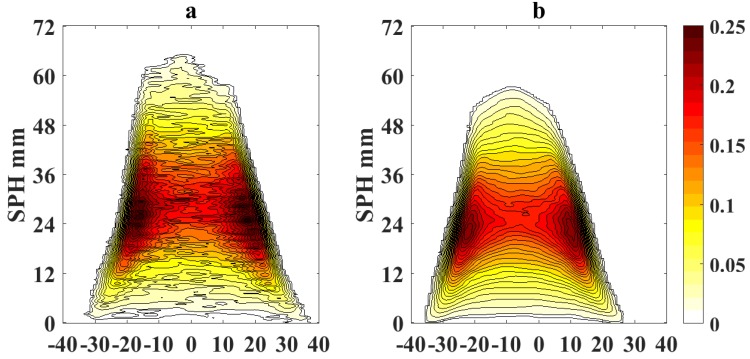
Magnified flame images for soot propensity analysis at SPH and OI = 21% (**a**) EVM; (**b**) PVM.

**Figure 6:**
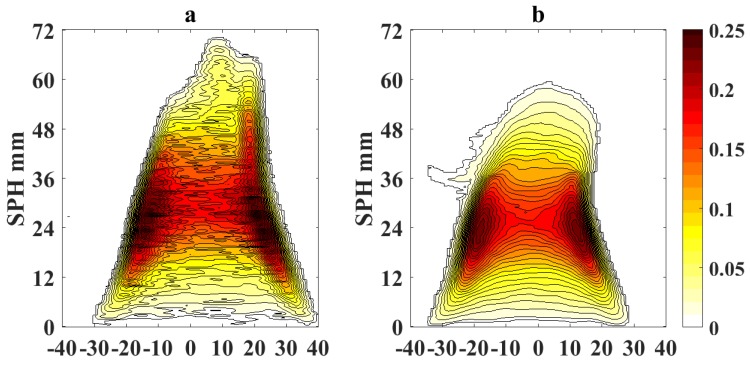
Magnified flame images for soot propensity analysis at ASPH and OI = 21% (**a**) EVM; (**b**) PVM.

Figure 5 and Figure 6 show the processed flame images at OI = 21% for a non-sooting condition when SPH is reached and for a sooting condition when SPH is surpassed, respectively. Color scale defines the normalized intensity of the flame images.

**Figure 7:**
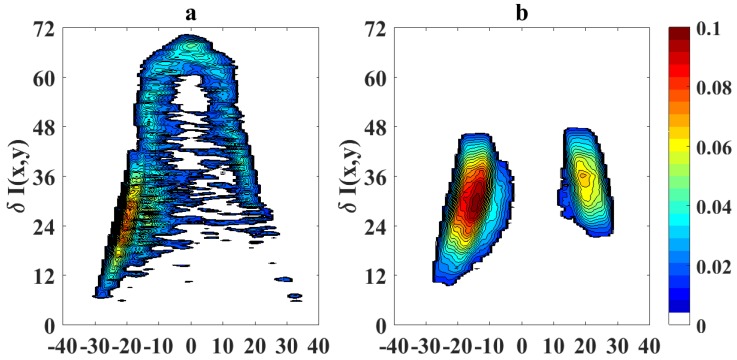
Pixel intensity difference δI(k,x,y) for magnified flame images at OI = 21% and SPH (**a**) EVM; (**b**) PVM.

**Figure 8:**
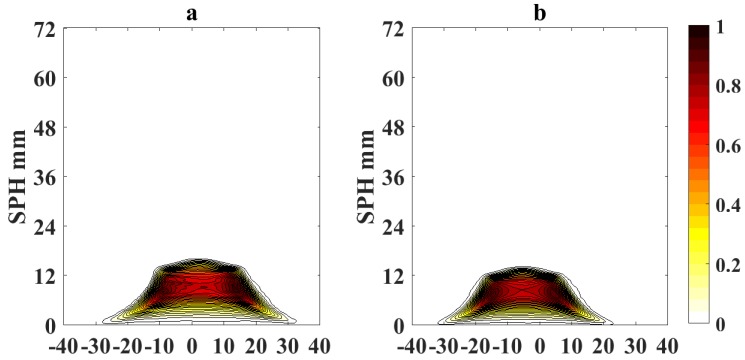
Magnified flame images for soot propensity analysis at SPH and OI = 35% (**a**) EVM; (**b**) PVM.

**Figure 9:**
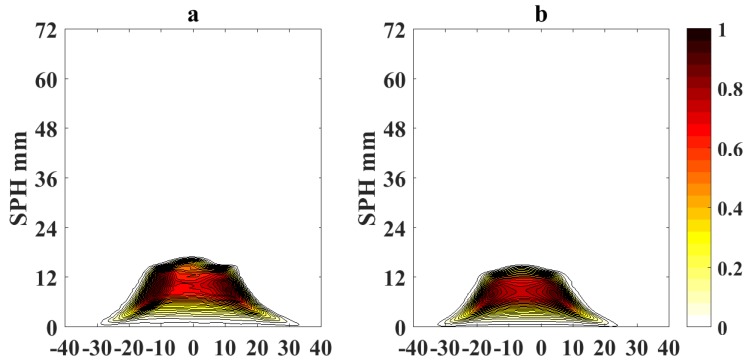
Magnified flame images for soot propensity analysis at ASPH and OI = 35% (**a**) EVM; (**b**) PVM.

**Figure 10:**
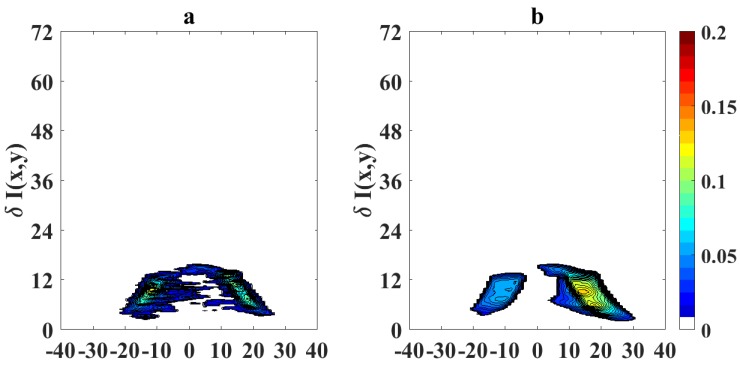
Pixel intensity difference δI(k,x,y) for magnified flame images at OI = 35% and SPH (**a**) EVM; (**b**) PVM.

To quantify the effects of using video magnification, we calculate a differential image corresponding to the pixel difference $\delta I\left(x,y\right)=|I_{original}\left(x,y\right)-I_{VM}\left(x,y\right)|$, where $I_{original}\left(x,y\right)$ represents the pixel intensity of the original flame image measured with the camera and $I_{VM}\left(x,y\right)$ is the pixel intensity of the magnified image. Figure 7 shows the absolute values of the pixel differences when the sooting condition of SPH is reached as in Figure 5 (OI = 21%). Following a similar scheme, Figure 8 and Figure 9 show the processed flame images at OI = 35%, for the different conditions respect to the SPH, and Figure 10 shows the absolute values of the pixel differences when the sooting condition of SPH is reached as in Figure 8. The plots of the pixel difference between amplified and original image clearly show that EVM produces a stronger magnification of the flame tip than PVM for the same amplification factor of $\alpha_{0}=20$.

**Figure 11:**
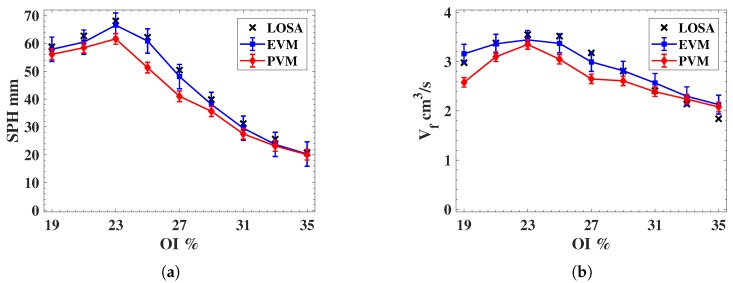
(**a**) Smoke point height as function of the oxygen index OI. (**b**) Fuel flow at smoke point height as function of the oxygen index OI.

Figure 11a,b present a soot propensity analysis in terms of the SPH and volumetric fuel flow rate at the SPH $V_{f}$, respectively. The analysis is presented as a function of the OI, which defines the experimental testing condition summarized in Table 1. To estimate the uncertainty of the analysis in terms of SPH and $V_{f}$, 100 samples are taken to obtain the average and variance of each indicator. These plots also present the referential values of SPH and $V_{f}$ obtained through the LOSA method by β, as described in Figure 4. It is clear that the EVM method yields results which are closer to the reference LOSA values. Note that the error bars of the EVM method are larger than those of the PVM method, which can be explained by the amplification of the input noise which is carried out by the EVM analysis.

### 5.3. Analysis and Discussion

Figure 5 and Figure 8 show the results of the application of video magnification to an image of a flame that has reached the SPH at OI = 21% and OI = 35%. Through simple direct visual inspection, We observe that EVM provides a stronger enhancement of relevant morphological changes when the flame tip begins to open (see Figure 5a and Figure 8a) compared to PVM for the same amplification factor. Similarly, experimental results for ASPH conditions (Figure 6 and Figure 9) show that EVM is again more effective than PVM when enhancing the morphological characteristics when the flame tip is open and soot is being released. The quantitive analysis of the differences between the original and amplified images presented in Figure 7 and Figure 10 clearly shows that EVM has a larger intensity difference around the tip of the flame, while PVM produces greater amplification at mid-flame height. In this context, EVM produces more useful results than PVM in terms of facilitating the identification the operational conditions of SPH and subsequently $V_{f}$ in a more precise way.

One problem with using EVM is that it relies in a linear amplification of subtle changes between consecutive images, and therefore amplifies the noise in the image. Consequently, images amplified through EVM tend to have more noise than the ones with PVM. From our experiments, we observed that the following factors affect the accuracy of the procedures for video magnification: With respect to uncertainties identified in the video magnification process, we observe that the following factors affect the accuracy of the procedures evaluated in our experiments:

- Lighting: measurements for soot propensity consider the same light applied to the CCD camera, corresponding to the flame chemiluminescence. Then, both EVM and PVM amplify pixel intensities that cannot be observed by the naked eye in the original flame image.
- Camera angle and position: If the camera angle and position are not fixed when capturing the sequence of flame images, movements from the camera can generate false image variations that will be added to the video magnification results [42]. This can seriously affect the accuracy of the SPH detection.
- Noise: measurements using optical instruments are subject to additive random variables related to the spurious noise and noise factor according to the camera type: CCD, ICCD or EMCCD [52]. Also, we can consider the presence of electronic noise on the video magnification block when the electronic devices are exposed to external perturbations.

The setup for our laboratory scale experiments considered configurations that reduced the sources of added noise in the captured videos of the flame under observation. Still, to reduce potential bias from remaining noise sources, we performed uncorrelated experiments to obtain average values of SPH and $V_{f}$. However, in industrial settings it may not be possible to completely eliminate these sources of noise, posing an extra challenge in the application of these techniques in practical combustion systems.

From SPH results obtained according to the experimental conditions described in Table 1, we observe that EVM presents better than PVM, in terms of its accuracy with respect to the target soot propensity analysis by LOSA. Unfortunately, a slightly larger uncertainty is obtained with EVM with respect to PVM, because of the inherent noise amplification of EVM.

When performing a quantitative evaluation of the uncertainty analysis for SPH, we obtained the following results:

(i) Average root-mean-square-error of EVM with respect to LOSA, $RMSE({\bar{SPH}}_{EVM})=5.1$ mm equivalent to 8%. This is more accurate than the average root-mean-square-error of PVM $RMSE({\bar{SPH}}_{PVM})=17.6$ mm equivalent to 25%.
(ii) Maximum uncertainty Δ (calculated for 95% confidence interval) for EVM and PVM are $\Delta (SPH_{EVM})=0.34$ mm and $\Delta (SPH_{PVM})=0.24$ mm and equivalent to $\Delta (SPH_{EVM})=0.55\%$ and $\Delta (SPH_{PVM})=0.36\%$, respectively.

For $V_{f}$ results, EVM also reveals a slightly larger uncertainty than the PVM method because of the noise amplification:

(i) Average root-mean-square-error of EVM $RMSE({\bar{V_{f}}}_{EVM})=0.48$ cm^3^/s equivalent to 14%, more accurate compared to the average root-mean-square-error of PVM with respect to LOSA $RMSE({\bar{V_{f}}}_{PVM})=0.94$ cm^3^/s equivalent to 26%.
(ii) Maximum uncertainty Δ (calculated for 95% confidence interval) for EVM and PVM are $\Delta (V_{fEVM})=0.08$ cm${}^{3}$/s and $\Delta (V_{fPVM})=0.06$ cm${}^{3}$/s and equivalent to $\Delta (V_{fEVM})=2.25\%$ and $\Delta (V_{fPVM})=1.59\%$, respectively.

With the overall uncertainty analysis, we verify that sooting propensity analysis by EVM presents an improved average performance, in terms of the SPH and $V_{f}$ results, in comparison to the results obtained through PVM. Concerning the random error for SPH and $V_{f}$, we observe that slightly smaller values are obtained with PVM, because of the noise amplification of EVM.

## 6. Conclusions

This paper presents novel methods for soot propensity analysis of combustion processes based on the amplification of the morphological changes recorded in a video sequence of a flame. The evaluated methodologies are based on EVM and PVM methods for video magnification. The results show that both techniques are able to magnify subtle changes in flame morphology that occur at the SPH, which facilitates detecting the point at which the flame starts releasing particulate matter.

Results of soot propensity analysis performed using EVM and PVM are benchmarked with reference results obtained through LOSA. The proposed techniques report acceptable average and random error, taking as key metrics for the evaluation the smoke point height (SPH) and volumetric fuel flow rate $V_{f}$ where SPH is reached. EVM presents the best results in terms of average error, but slightly higher random uncertainty with respect to PVM results, mainly given by the inherent amplification of noise of the EVM technique.

The results of this work are encouraging, showing the feasibility of performing soot propensity analysis for detecting emissions of particulate matter to the environment using flame images. The proposed technique can overcome the intrinsic delay of direct measurements of PM emissions in industrial settings using gas chromatographs. Moreover, the main component of the methods is an image acquisition device that may be already present in industrial settings for monitoring purposes. This makes it a simpler and cheaper alternative to LOSA, which requires expensive equipment that is normally unsuitable for conventional industrial combustion facilities. Nevertheless, there are still several practical challenges to be solved, including accounting for sources of signal noise in industrial settings and working with turbulent, unsteady flames which are typically found in combustion chambers.

From previous results, unified behavioral analysis for axisymmetric or co-flow diffusion flames was proposed for different fuels [11]. This analysis concerns the smoke point height (SPH), volumetric fuel flow rate $V_{f}$ where SPH is reached, and soot volume fraction within the flame β, when the oxygen index, flow conditions and the dominance of soot formation over oxidation varying. Then, this model-free soot propensity analysis approach based on video magnification could be used for different fuels, because it is strictly based on measurement conditions of flame images, and β by LOSA as reference.

Future work considers the detailed characterization of the computational cost for the processing flow based on EVM, and the deployment of a prototype as an embedded platform for real-time estimations to be tested in both laboratory scale experiments and industrial settings. The final objective is to validate our findings and evaluate their effective utility on the development of novel instrumentation for cleaner combustion processes.

## Figures and Tables

**Table 1:** Experimental conditions.

OI (%)	$Q_{\mathrm{air}}$ (slm)	$Q_{O_{2}}$ (slm)	$Q_{N_{2}}$ (slm)	$Q_{\mathrm{total}}$ (slm)	$m_{C_{2}H_{4}\min}$ (cm^3^/s)	$m_{C_{2}H_{4}\max}$ (cm^3^/s)
19%	-	17.1	72.9	90	2.31	4.31
21%	90	-	-	90	2.71	4.71
23%	87.8	2.2	-	90	2.88	4.89
25%	85.4	4.6	-	90	2.85	4.85
27%	83.2	6.8	-	90	2.51	4.51
29%	80.8	9.2	-	90	2.14	4.14
31%	78.7	11.3	-	90	1.74	3.74
33%	76.3	13.7	-	90	1.48	3.48
35%	74	16	-	90	1.33	3.17

## Abbreviations

The following abbreviations are used in this manuscript:

VM	Video Magnification
EVM	Eulerian Video Magnification
PVM	Phase Video Magnification
SPH	Smoke Point Height
LOSA	Line-Of-Sight Attenuation
$S_{\mathrm{for}}$	soot formation rate
$S_{\mathrm{ox}}$	soot oxidation rate
RMSE	Root-mean-square-error
